# Ex Vivo Conjunctival Retention and Transconjunctival Transport of Poorly Soluble Drugs Using Polymeric Micelles

**DOI:** 10.3390/pharmaceutics11090476

**Published:** 2019-09-14

**Authors:** Silvia Pescina, Leticia Grolli Lucca, Paolo Govoni, Cristina Padula, Elena Del Favero, Laura Cantù, Patrizia Santi, Sara Nicoli

**Affiliations:** 1 Department of Food and Drug, University of Parma, Parco Area delle Scienze 27/A, 43124 Parma, Italy; silvia.pescina@unipr.it (S.P.); cristina.padula@unipr.it (C.P.);; 2 Department of Medicine and Surgery, University of Parma, via Volturno 39, 43126 Parma, Italy; paolo.govoni@unipr.it; 3 Department of Medical Biotechnologies and Translational Medicine, LITA, University of Milan, 20090 Segrate (MI), Italy; elena.delfavero@unimi.it (E.D.F.); laura.cantu@unimi.it (L.C.)

**Keywords:** ocular delivery, polymeric micelles, conjunctiva, small angle X-ray scattering (SAXS), econazole, cyclosporine, dexamethasone, solubility, TPGS, poloxamer 407

## Abstract

This paper addresses the problem of ocular delivery of lipophilic drugs. The aim of the paper is the evaluation of polymeric micelles, prepared using TPGS (d-α-Tocopheryl polyethylene glycol 1000 succinate), a water-soluble derivative of Vitamin E and/or poloxamer 407, as a vehicle for the ocular delivery of dexamethasone, cyclosporine, and econazole nitrate. The research steps were: (1) characterize polymeric micelles by dynamic light scattering (DLS) and X-ray scattering; (2) evaluate the solubility increase of the three drugs; (3) measure the in vitro transport and conjunctiva retention, in comparison to conventional vehicles; (4) investigate the mechanisms of enhancement, by studying drug release from the micelles and transconjunctival permeation of TPGS; and (5) study the effect of micelles application on the histology of conjunctiva. The data obtained demonstrate the application potential of polymeric micelles in ocular delivery, due to their ability to increase the solubility of lipophilic drugs and enhance transport in and across the conjunctival epithelium. The best-performing formulation was the one made of TPGS alone (micelles size ≈ 12 nm), probably because of the higher mobility of these micelles, an enhanced interaction with the conjunctival epithelium, and, possibly, the penetration of intact micelles.

## 1. Introduction

The treatment of ocular diseases is a challenging task, due to the peculiar structure of the eye, characterized by the presence of several anatomical and functional barriers. Topical drug application, i.e., drug instillation in the conjunctival sac or on the ocular surface, is characterized by a very short residence time, possible systemic absorption, and difficult drug penetration into the ocular tissues. The consequence is a low ocular bioavailability both in the anterior eye segment (eye surface, cornea, conjunctiva, anterior and posterior chambers, iris), and, even more, in the posterior eye segment. Colloidal formulations are increasingly investigated to overcome some of the cited limitations and, among them, polymeric micelles have been shown to be able to provide efficient drug delivery. These nanocarriers (10–200 nm), simple to prepare and easily sterilized, are self-assembled aggregates of amphiphilic polymers with a nonoily hydrophobic core and a hydrophilic corona. Besides their solubilizing properties, micellar formulations are also reported to prolong the contact time with ocular tissues, increase the stability of therapeutic proteins and peptides, and enhance transmembrane transport with different mechanisms [1,2]. Indeed, recent data have demonstrated the ability of polymeric micelles to increase drug uptake and permeation across different ocular tissues [2,3] such as the cornea [4,5], sclera [6,7], and conjunctival cells [8,9]. Due to these advantages, interest in the field has increased over time and brought about, in 2018, the approval by the FDA of a cyclosporine-based micellar product for ophthalmic use. The interest in these formulations is also shown by the large number of recent scientific publications [2,10,11,12,13]. Polymeric micelles made by polyvinyl caprolactam–polyvinyl acetate–polyethylene glycol graft copolymer were prepared to increase the solubility of fluorometholone, a corticosteroid used for the treatment of inflammation of the anterior segment of the eye, and to promote its transcorneal diffusion in an ex vivo model [5]. Triamcinolone acetonide was formulated in hyaluronic acid‒hexadecylamine derivatives-based micelles [14], and in hydrogenated castor oil-60/Octoxynol-40 mixed micelles [15]. Cyclosporine, a poorly soluble immunosuppressant cyclic peptide, has been the subject of several studies involving micelles [2]; among them, the use of polyoxyl 35 hydrogenated castor oil micelles demonstrated, in a dry-eye syndrome model, the improvement of the functionality and morphology of the conjunctival epithelium [16]. 

**Figure 1:**
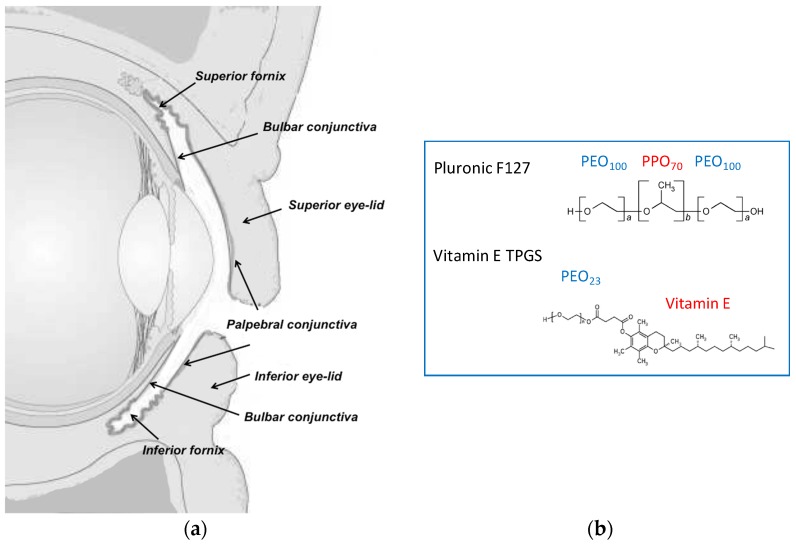
(**a**) Scheme of the vertical section of the eye. Reprinted with permission from Pescina et al. [29]. Copyright Taylor & Francis, 2017. (**b**) Chemical structure of the polymeric surfactants used in the present work.

The conjunctiva is the mucosal membrane that covers the internal part of the eyelids and the visible sclera, and ends at the corneoscleral limbus (Figure 1). The conjunctiva is essential to maintain the homeostasis of the ocular surface by protecting the underlying tissues, producing the aqueous and mucus layers of the tear film, providing immunological defense, and facilitating globe movement [17]. The barrier function of this epithelium is mainly due to the presence of tight junctions in the apical layer, even though the intercellular spaces are wider in comparison to the corneal epithelium [1] and the permeability is 8.6 ± 4.4 fold higher [18]. The conjunctiva can represent the site of diseases (allergic and infectious conjunctivitis, inflammation, dry eye syndrome), but also represents the first barrier to posterior segment targeting: once the bulbar conjunctiva is crossed, the drug can diffuse through the sclera into the uvea and RPE (retinal pigmented epithelium), to possibly reach the neural retina and the vitreous humor [19,20]. Additionally, it is important to underline that blood and lymphatic vessels, present in the conjunctival tissue, are responsible for the unavoidable systemic absorption [18,21,22], occurring in particular across the palpebral conjunctiva. For these reasons, drug retention in the conjunctival tissue and transconjunctival transport are both relevant for the understanding of drug bioavailability.

The aim of this paper is the evaluation of polymeric micelles prepared with TPGS (d-α-Tocopheryl polyethylene glycol 1000 succinate), a water-soluble derivative of Vitamin E and/or poloxamer 407 (structure in Figure 1b) as vehicle for the ocular delivery of lipophilic drugs. These polymers were selected on the basis of their solubilization, mucoadhesion and penetration enhancing properties [7]. Furthermore, the enzymatic release of vitamin E and vitamin E succinate from TPGS [7], mediated by ubiquitous esterases, may provide additional antioxidant properties. Finally, the thermoreversible properties of poloxamer 407 increase the viscosity of the system, thus enhancing drug retention on the ocular surface. The drugs selected were dexamethasone, cyclosporine, and econazole nitrate. Cyclosporine and dexamethasone are topically administered for the treatment of different inflammatory diseases affecting the surface of the eye, but they are also successfully used in the treatment of posterior eye diseases [6,23,24]. Econazole is used primarily for the treatment of mycosis of the surface of the eye [25,26,27]; additionally, some researchers provided the proof of principle of a potential application in reducing vascular loss and progression of age-related macular degeneration (AMD) [28]. 

The research steps were (1) to characterize polymeric micelles by DLS and X-ray scattering; (2) to evaluate the solubility increase of the three drugs; (3) to measure the in vitro transport and conjunctiva retention, in comparison to conventional vehicles; (4) to investigate the mechanisms of enhancement, by studying drug release from the micelles and transconjunctival permeation of TPGS; and (5) to study the effect of micelles application on the histology of conjunctiva. 

All transport and permeation experiment were performed in vitro using freshly excised porcine conjunctiva.

## 2. Materials and Methods

### 2.1. Materials

Cyclosporine (MW 1202.61 g/mol, LogP:3.35 [30] and dexamethasone (MW 392.46 g/mol, logP:1.87) were purchased from Alfa Aesar (Kandel, Germany). Poloxamer 407 (Pluronic^®^ F127 MW 12.6 kDa) was a kind gift from BASF (Ludwigshafen, Germany). d-α-Tocopheryl polyethylene glycol 1000 succinate (TPGS, MW 1513 g/mol) was a kind gift from PMC ISOCHEM (Vert-Le-Petit, France), Miglyol^®^ 810 (caprylic/capric triglycerides), was from Degussa-Hüls AG (Frankfurt, Germany) and Transcutol^®^ was a kind gift from Gattefossé (Lyon, France). Trifluoroacetic acid (TFA, MW 114.02 g/mol), and bovine serum albumin (BSA) were purchased from Sigma Aldrich (St. Louis, MO, USA). Econazole nitrate (Sifavitor, Milan, Italy, MW 381.68 g/mol, econazole logD_7.4_: 5.44) was a kind gift from Angelini (ACRAF, Roma, Italy). Pure water (Purelab^®^ Pulse, Elga Veolia, UK) was used; all other chemicals were of analytical or HPLC grade.

Buffered solutions used were phosphate-buffered saline (PBS; 0.19 g/L KH_2_PO_4_, 5.98 g/L Na_2_HPO_4_·12H_2_O, 8.8 g/L NaCl, pH 7.4), acetate buffer 0.1 M pH 4.0 (1.86 g/L CH_3_COONa, 4.42 mL/L CH_3_COOH), acetate buffer 0.1 M pH 5.8 (1.476 g/L CH_3_COONa, 4.674 mL/L CH_3_COOH, to pH 5.8 with NaOH).

### 2.2. Micelle Preparation

Blank micelles containing only TPGS (T), only poloxamer 407 (P), or both polymers in a 50:50 (TP) or 25:75 (PT) molar ratio were prepared. The exact composition is reported in Table 1. Micelles were prepared using the dissolution method. Briefly, for T preparation, TPGS was accurately weighted and added to 5 mL of water under stirring, until complete dissolution. For P preparation, 5 mL of water were immersed in an ice bath and poloxamer 407 was added under stirring. The limpid micellar solutions obtained were further stirred (either at room temperature or at 4 °C) for additional 30 min. TP and PT were prepared either by dissolving both powders in water or by mixing volumes of T and P containing equal moles of poloxamer and TPGS (TP) and by stirring in an ice bath for at least 30 min.

Cyclosporine, dexamethasone, and econazole were loaded by adding an excess of each drug to the prepared blank formulations. The obtained suspensions were stirred for 24 h at either 25 °C (T, TP) or 4 °C (P and PT). Drug-loaded micelles were separated from undissolved drug by centrifugation at 12,000 rpm for 10 min at either 25 °C (T, TP) or 4 °C (PT, P). In these conditions, the drug loading corresponds to its solubility. 

### 2.3. Micelle Characterization: Dynamic Light Scattering and SAXS Analysis

Micelles were characterized in terms of pH, size, and zeta potential. 

To determine the micelle size, samples were analyzed after 1:10, 1:50, or 1:100 dilution in water at 25 °C, with an incidence angle of 173° (dispersant refractive index 1.33; viscosity 0.89 mPa·s), using a Zetasizer Malvern (Ver. 7.12, Malvern Instruments LTD., Malvern, Worcestershire, UK). Size distribution was reported by intensity. 

Zeta potential was measured with the same instrument; blank samples were diluted 1:10, 1:20, or 1:40 with 0.5 mM KCl before analysis. The 1:40 dilution was then selected due to the better quality of the measurements obtained. 

Small-angle X-ray scattering (SAXS) experiments were carried out on an ID02 instrument at ESRF (Grenoble, France). The incident wavelength was 0.1 nm, with a sample to detector distance of 1 m, which provided a range of scattering vectors 0.09 < *q* < 6 nm^−1^. Blank micelles were put in plastic capillaries (ENKI, Concesio, Italy) of 2 mm path length, controlling the temperature using an external thermostat. The duration time of single exposures was kept very short (0.1 s) and different short frames are averaged. All scattering data were first checked for radiation damage, normalized, and background-corrected. Data analysis was performed using SasView 4.2.1 software [31]. The scattering curve from the TPGS solution was fitted to a core-shell sphere model combined with a hard-sphere (HS) structure factor (3% volume fraction). For the polydispersity of the core, a Schulz distribution was chosen. For poloxamer micelles, the positions of Bragg peaks were recorded to define the symmetry of the liquid‒crystalline structure and the dimensions of the primitive cell.

### 2.4. Drug Solubility Studies

Excess amounts of cyclosporine, dexamethasone, or econazole nitrate were separately added to blank micelles (Table 2). After stirring for 24 h, 100 µL were sampled and centrifuged at 12,000 rpm for 10 min at either 25 °C (T, TP) or 4 °C (PT, P). Controls were set up to evaluate the impact of the centrifugation temperature on the final solubility. The supernatant was sampled, appropriately diluted, and analyzed by HPLC-UV (methods in Table 3). 

Further solubility samples were collected up to 30 (econazole nitrate and dexamethasone) or 36 (cyclosporine) days to evaluate the time dependence of solubility.

### 2.5. Tissues 

Porcine eyeballs were obtained from 10–11-month-old animals (breeds: Landrance and Large White, weight 145–190 kg) from a local abattoir (Macello Annoni S.p.A., Parma, Italy). Eyeballs were transported to the laboratory immersed in PBS at 4 °C, and conjunctival tissue was used within 2–3 h from enucleation. Conjunctival thickness was measured at the fornix by means of a digital caliper (0.001 mm resolution; Absolute Digimatic 547-401, Mitutoyo, Milan, Italy). 

### 2.6. Transconjunctival Permeation Studies 

**Figure A1:**
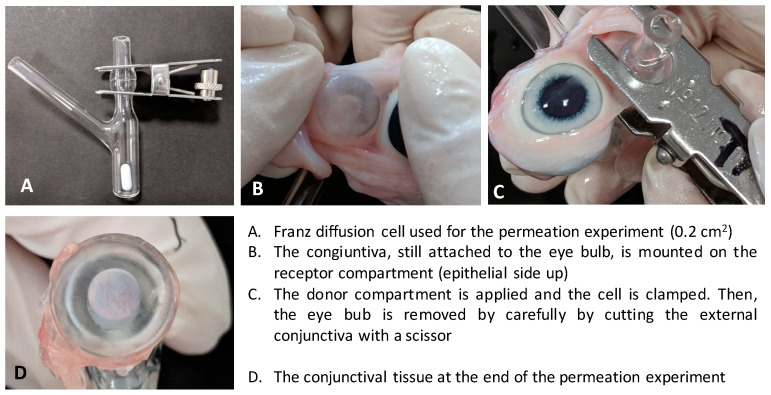
Setup of the transconjunctival permeation experiment. (**A**) Franz diffusion cell used for the permeation experiment (0.2 cm^2^); (**B**) The conjunctiva, still attached to the eye bulb, is mounted on the receptor compartment (epithelial side up); (**C**) The donor compartment is applied and the cell is clamped. Then, the eye bub is removed by carefully cutting the external conjunctiva with a scissor; (**D**) The conjunctival tissue at the end of the permeation experiment.

Experiments were performed using Franz-type diffusion cells (area: 0.2 cm^2^). The conjunctiva was isolated from the inferior part of the eyeball and, still attached to the eyeball, was mounted on the diffusion cell lower compartment, with the stromal side facing the receptor compartment, taking care to avoid tissue stretching. Then, the upper compartment was applied, the system was clamped, and the eyeball was carefully removed by cutting the external conjunctiva with a scissor. The method of conjunctival mounting and cell assembly is illustrated in Appendix A (Figure A1).

After cell assembly, the lower compartment was filled with approximately 4 mL of degassed receptor solution. The cell was placed in a thermostatic bath at 37 °C; the receptor solution was magnetically stirred, to avoid any boundary layer effects. The donor compartment was filled with 80 µL of formulation and covered with parafilm. Every hour, the receptor fluid was sampled and analyzed. Details on the receptor phase, donor phase, and donor concentrations of each drug are reported in Table 2. Due to the low water solubility of the compounds tested, in some cases it was necessary to add TPGS or BSA as a solubilizing agent to the receptor solution to ensure sink conditions. The solubility of each drug in the receptor phase is reported in the footnotes of Table 2. In the case of formulations T with dexamethasone, the transconjunctival transport of TPGS was evaluated as well. Experiments were replicated three to seven times. 

The amount of drug permeated, normalized for the permeation area, was plotted against time. Because the steady state was never reached, the instantaneous flux *J* (µg/cm^2^ h) was calculated between the 4th and the 5th hour. Then, the apparent permeability coefficient *P* (cm/s) was calculated as *P* = *J*/*C*d, with *C*d (µg/mL) being the concentration of the drug in the donor chamber.

At the end of the experiment (5 h) the formulation was removed and the conjunctiva was washed (saline solution, 5 mL), carefully dried with filter paper, and transferred to a vial. Drugs retained in the tissues were extracted overnight with 1 mL of mixture (Table 2). Extraction solutions were filtered with 0.45 µm regenerated cellulose filters (Phenomenex, Torrance, CA, USA) before HPLC analysis. The extraction procedures were previously validated and the percentage recovery (%), determined by computing the ratio of the amount of drug extracted from spiked tissue to the amount of drug added, was included between 94% and 100% (Table S1). 

### 2.7. Conjunctiva Histology

For histological analysis, samples of conjunctiva, both freshly explanted and after treatment at 37 °C for 5 h with the different blank vehicles reported in Table 2, were fixed in 10% formaldehyde. Subsequently, the samples were embedded in paraffin and sectioned using a microtome. Staining was carried out using Harris hematoxylin/eosin. Images were taken using an optical microscope Nikon Eclipse 80i, equipped with a camera Nikon Digital Sight DS-2Mv and connected to the control software, NIS Elements F (Nikon Instruments, Calenzano, Italy). 

### 2.8. Dexamethasone Release from Micelles

For dexamethasone release studies, a cellulose dialysis tube (flat width 10 mm; MWCO 14,000 Da; Sigma Aldrich) was used. First 0.2 mL of drug-loaded micelles (T and P) or drug solution (concentration 0.5 mg/mL; solvent ethanol:PBS 50:50) was inserted into the dialysis tube (length: 4.5 cm). The tube was sealed at both ends and immersed in 45 mL of buffer solution (PBS pH 7.4) thermostated at 37 °C and magnetically stirred (250 rpm). After 0.5, 1, 2, 3, 4, 5, and 6 h, 300 µL of the external solution were sampled and analyzed by HPLC. The solubility of dexamethasone in PBS (79 ± 4 µg/mL) ensured sink conditions.

### 2.9. HPLC-UV Methods

Analyses were performed in isocratic conditions using an HPLC-UV system (Infinity 1260, Agilent Technologies, Santa Clara, CA, USA) and a guard column C_18_ 3.2 × 0.8 mm (Security Guard™, Phenomenex). The injection volume was 100 µL. Details on the HPLC methods used for the different compounds are reported in Table 3. Parameters related to method validation are reported in Table S1. 

### 2.10. Statistical Analysis

The significance of the differences between values was assessed using one-way ANOVA followed by the Bonferroni test (Kaleidagraph 4.5.2). Differences were considered statistically significant when *p* < 0.05. In the text and figures, all data are reported as the mean value ± standard deviation. 

## 3. Results and Discussion 

Drug transport across the conjunctiva has a crucial role because it mediates the delivery to the posterior segment, following the conjunctival‒scleral route [19], but also the nonproductive and undesirable systemic absorption (taking place mainly across the palpebral conjunctiva) [18,21].

The comprehension of the permeation kinetics across this layer is thus very important in the design of drug delivery systems, especially in the presence of excipients that act as permeation enhancers or increase the permanence time on the ocular surface. In contrast with the permeation across the cornea, limited permeation data can be found in the literature across rabbit [1,32,33,34,35,36,37], bovine [38], or porcine [39] isolated conjunctiva. Most papers studied the effectiveness and safety of ophthalmic formulations using conjunctival epithelial cells layers or in vivo models. However, ex vivo models represent a valuable tool for the understanding of diffusive phenomena across the conjunctiva [39,40], as well as for the evaluation of ophthalmic formulations, particularly for the study of permeation enhancers [36] and drug partitioning between the delivery systems and the tissue [38].

Among colloidal systems, polymeric micelles can be very useful to solubilize hydrophobic drugs, to increase the retention on ocular surface and enhance transport across epithelia [2,41], even though some authors reported, for certain polymeric surfactants, a strong permeability-depressing effect [42].

### 3.1. Tissue Characterization

**Figure A2:**
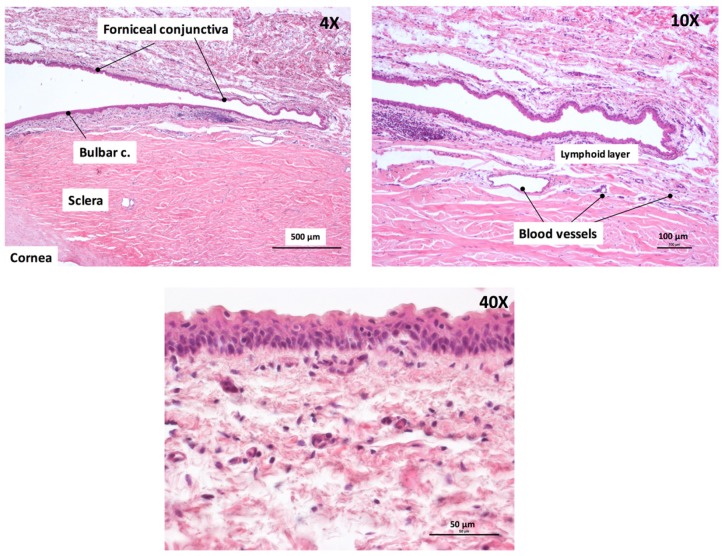
Histology of porcine conjunctiva at different magnifications.

All permeation experiments were performed using the fornix conjunctiva in close contact with the bulbar conjunctiva. The choice was determined by the easier isolation and manipulation of this part and to the limited extension of the bulbar conjunctiva in porcine eyes. Furthermore, the literature data indicate a comparable permeability of different parts of the conjunctiva [1,43], despite a different number of cell layers [17]. The thickness of the tissue used was measured with a digital caliper and was between 200 and 400 µm. The variability of tissue thickness is related to the different thickness of the stroma. Details on the procedure and histology of the freshly explanted conjunctiva used in the present research are reported in Appendix A (Figure A1 and Figure A2).

### 3.2. Characterization of Blank Micelles

Micelles appear as colorless and transparent formulations; T and TP were characterized by low viscosity, while P formulation, due to the thermosensitive characteristics of poloxamer 407, is a gel having a viscosity of approximately 490 Pa·s at 25 °C and 765 Pa·s at 37 °C (shear rate: 0.5 1/s), and a convenient shear thinning behavior [44]. The pH of blank micelles was around 6–7 (Table 4). 

#### 3.2.1. Light Scattering: Size and Zeta Potential

For the determination of the size, micelles were diluted 1:10, 1:50, and 1:100. The size obtained using the different dilutions was comparable; 1:10 was chosen due to the higher quality of the measures obtained. T micelles were monodispersed (Table 4) with an average size of 14 nm. P micelles (poloxamer 407 was the only polymer) were highly polydisperse and the collected data were poorly reproducible (Table S2), even after a 1:100 dilution, when micelle‒micelle interactions should be prevented. Interestingly, the addition of TPGS to poloxamer (1:1 molar ratio), to obtain TP, determined the formation of a monodispersed system, with an average micelle size of 20 nm (Table 4). This result suggests a strict interaction between the two polymers and the formation of mixed micelles. 

The zeta potential of the micelles, slightly negative, was close to zero (Table 4), in agreement with the neutral nature of the polymers used. 

#### 3.2.2. X-ray Scattering

SAXS experiments were carried out to describe in more detail the structure of micelles, and the analysis was performed on the undiluted formulations. 

**Figure 2:**
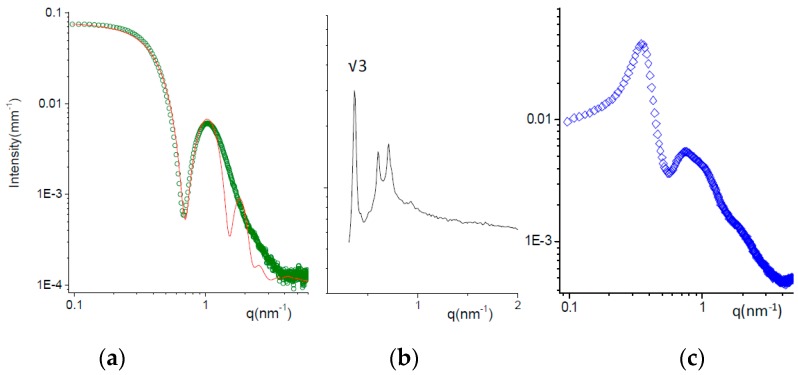
SAXS spectra of micellar solutions at *T* = 25 °C. (**a**) Formulation T (green dots); fitting curve (red line) obtained with a core-shell-sphere model for the micellar form factor combined with a hard-sphere structure factor. (**b**) Formulation P (black line). The position of characteristic peaks are reported in the Supplementary Materials. The ratio between the first observed peak and the first order peak position *q* * is reported. The ordered phase is a face-centered cubic (FCC) micellar phase. (**c**) Formulation TP (50:50 mole ratio).

Figure 2a shows the scattering of the formulation T at 25 °C. The intensity profile has been fitted to a core shell sphere model combined with a hard sphere (HS) structure factor, as reported by Puig-Rigall et al. [45]. TPGS micelles have a central core, 3 nm radius, with low polydispersity, on the order of 0.05. The radius of the core corresponds to the length of the extended tocopherol‒succinate molecule, indicating no interdigitation of the tails. The hydrophobic core is surrounded by a highly hydrated shell with a thickness of ca. 2.2 nm. The total size of the micelles (10.4 nm) is in good agreement with the hydrodynamic diameter assessed by DLS (13.8 nm; see Table 4), with the hydrodynamics being more affected by the highly hydrated terminals of polyethylene chains.

The SAXS intensity spectrum of formulation P at 25 °C is reported in Figure 2b. A liquid crystalline phase is observed, with characteristic Bragg peaks (the q positions are reported in Table S3). The crystalline phase can be identified by analyzing the ratio between the position of the peaks over the value of the first-order peak position *q* * corresponding to the Bravais Lattice parameter. The ratio *q*_i_/*q* * is reported in Figure 2 for the first peak observed. The phase has been identified as a face-centered cubic (FCC), typical for poloxamer 407 micelles between 20% and 40% concentration at room temperature [46,47,48]. The size of poloxamer 407 micelles, ordered in the cubic lattice, was about 13 nm. 

Mixed micelles TP (50:50 mole ratio) were prepared either by dissolving both powders in water or by mixing volumes of T and P containing equal moles of poloxamer and TPGS (TP). The comparison of the corresponding SAXS spectra is reported in Figure S1, showing very similar intensity profiles. The structural features of TP micelles can be inferred from the SAXS spectrum in Figure 2c. The intensity profile is modulated by the contributions of both the particle form factor, visible mainly in the high *q* region, and the solution structure factor, due to the intermicellar interactions. The form factor is typical for core shell structures, as expected for micelles. The structure factor oscillations, first peak at *q*_1_ = 0.35 nm^−1^, allow us to calculate the characteristic intermicellar distance *d* = 2π/*q*_1_ = 18 nm. With the volume fraction of the TP micelles being on the order of 12%, we can estimate the micellar volume (700 nm^3^) and thus its size = 11 nm. This value is smaller than the hydrodynamic size (20 nm), as already mentioned for TPGS micelles. The higher discrepancy between the two methods obtained in case of TP with respect to T may be due to the longer PEO chains in the poloxamer compared to TPGS. 

Structural results show that all systems self-aggregate in small micelles with a core shell structure; the presence of TPGS molecules helps the poloxamer with the formation of monodisperse spherical micelles. The size of the systems is in good agreement with that found in the literature for poloxamer 407 [49], TPGS [50,51] or mixed micelles [7], using microscopic techniques such as TEM or cryo-TEM. 

### 3.3. Cyclosporine

#### 3.3.1. Cyclosporine Solubility

**Figure 3:**
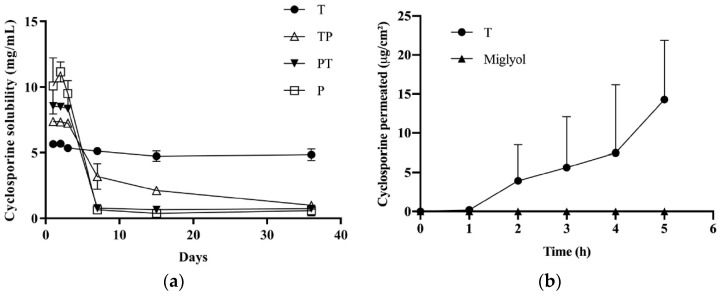
(**a**) Cyclosporine’s apparent solubility when incorporated in micelles of TPGS, poloxamer 407, and TPGS:poloxamer 407. (**b**) Transconjunctival permeation profile of cyclosporine from formulation T (*n* = 4) and Miglyol^®^ solution (drug conc. = 5 mg/mL; *n* = 3).

Formulation T increased the cyclosporine solubility from 0.035 mg/mL (water solubility) to about 5 mg/mL (Figure 3a); the solubility reached was maintained up to 36 days. In the presence of poloxamer 407, very high cyclosporine concentrations (up to 11 mg/mL) were obtained in the first three days, followed by drug precipitation and a final solubility lower than 1 mg/mL. This combination of rapid dissolution and supersaturation followed by a slow precipitation has been reported for several drugs and has been attributed to the transient stabilization of an amorphous form, followed by recrystallization and precipitation [52,53]. The tendency of poloxamer 407 to give rise to a supersaturated solution has been reported for other drugs such as curcumin [54], tacrolimus [55], and celecoxib [56]. Drug precipitation could also be due to different cyclosporine localization in the P micelle core due to the V-shaped form of PPO blocks during micelle formation, and to the looser packing of PPO hydrophobic chains. The higher solubilization power of TPGS compared to poloxamer 407 can be explained by the higher hydrophobicity of the TPGS micelle core, together with the high logP of cyclosporine (3.35). Furthermore, it is worth mentioning that the addition of poloxamer 407 to a saturated T solution determined the cyclosporine precipitation (data not shown), suggesting that poloxamer 407 can displace the drug from the hydrophobic core of T micelles, and confirming the interaction between the two polymers seen in the DLS. This also justifies the low cyclosporine solubility obtained in the case of TP and PT. 

In a previous paper, we described the preparation of micelles of poloxamer 407:TPGS (1:1 molar ratio) for cyclosporine delivery [7]; cyclosporine precipitation was not observed, probably because of the different preparation method, which involved the use of ethanol and brought to a 5% (*v*/*v*) ethanol concentration in the final formulation. 

Table 4 illustrates the characteristics of cyclosporine-loaded micelles. The size of T and TP micelles was unchanged with respect to blank micelles.

#### 3.3.2. Cyclosporine Permeation across the Conjunctiva 

Given that the only micellar formulation able to sustain cyclosporine solubility was T, this vehicle was used for transconjunctival permeation experiments. As a control, a 5 mg/mL cyclosporine solution in Miglyol^®^ was used. The results are reported in Figure 3b. By using Miglyol^®^ as a vehicle, no transconjunctival transport was observed and only traces (<LOQ) of cyclosporine were found in the tissue. This indicates that the conjunctiva represents a relevant barrier for cyclosporine transport and that the partition of the drug into the tissue (clearly hampered by the affinity between the lipophilic drug and Miglyol^®^) plays an important role. Another phenomenon that could have hindered cyclosporine transconjunctival permeation is the presence of efflux systems [57,58,59]. Indeed, it has been demonstrated that the transport rate of cyclosporine across cultured rabbit conjunctival epithelial cells was greater in the basolateral to apical direction than in the opposite direction [57]. Furthermore, the literature data indicate that these efflux systems are preserved in excised conjunctival tissues for at least 4 h [40].

The use of the micellar formulation determined a transconjunctival flux (calculated between the 4th and 5th hour) of 8.27 ± 4.67 µg/cm^2^h and an apparent permeability coefficient of (4.59 ± 2.59) × 10^−7^ cm/s, while the amount found inside the conjunctiva was 13.34 ± 15.92 µg/cm^2^ (Table 5). The reasons for polymeric micelles’ good performance could be: (1) the favorable partition; (2) the permeation-enhancing property of TPGS [60], which, dissociated from micelles, can intercalate between the phospholipids of the conjunctival cells, increasing membrane fluidity and permeability [61]; (3) the inhibition of efflux pump by TPGS [62]; (4) the penetration of intact micelles [2]; and, possibly, (5) the higher drug thermodynamic activity compared to the Miglyol^®^ solution. 

Both the permeation profile and the accumulation are characterized by high variability, as also reported by other authors evaluating many different drugs across bulbar/fornix porcine conjunctiva [39].

### 3.4. Econazole Nitrate

#### 3.4.1. Econazole Nitrate Solubility

Econazole is a basic compound characterized by a pKa (base) of 6.5 ± 0.4 [30]. The solubility of the nitrate salt in water is 1.57 ± 0.05 mg/mL and the saturated solution has a pH 4.25 ± 0.10; the % of unprotonated form at this pH is approximately 0.5% [30]. In the presence of polymeric micelles, the solubility increased to approximately 4 mg/mL, regardless of the micelle composition (Table 4). The similar solubilization capability of the two polymers suggests that econazole is located in the corona of micelles, made of PEO in all cases; this is also in agreement with the relatively high hydrophilicity of the molecule, which is more than 99% in ionized form. The size of the micelles was not affected by econazole loading, while the zeta potential was slightly increased to a few mV, in agreement with the positive charge of the drug (Table 4).

While the pH of TP and P formulations was higher than 4, acceptable for ocular instillation [63], it was necessary to increase the pH of T (3.36). The use of a PBS buffer at pH 7.4 as a vehicle for TPGS dissolution was discounted, since it caused a significant reduction in drug solubility (2.43 ± 0.53 mg/mL; pH = 5.06 ± 0.01). On the contrary, by adding NaOH 0.5M (approximately 80 µL/mL formulation) to formulation T, it was possible to obtain a pH of 5.1 without drug precipitation. However, at this pH value, the stability of the drug was poor, with the drug concentration being 1.78 ± 0.17 mg/mL after one month.

#### 3.4.2. Econazole Nitrate Retention in the Conjunctiva

All the formulations prepared were evaluated on the conjunctival tissue, and a drug dispersion in Miglyol^®^ (3.8 mg/mL) was used as the control vehicle. Econazole was not recovered in the receptor compartment before the fifth hour, and only in some replicates of formulation T (approximate concentration = LOQ). The absence of drug permeation was confirmed using two different receptor phases: PBS containing either TPGS 0.2 mM (econazole solubility 91 ± 8 µg/mL) or 1% BSA (econazole solubility 245 ± 29 µg/mL). Econazole nitrate extraction from the tissue highlighted the different behavior of the micelles tested, as reported in Table 5. In fact, despite the same drug loading, micelles made of TPGS alone (formulation T, both at pH 3.4 and 5.1) performed 4- to 5-fold better than the others. Miglyol^®^ dispersion gave the lowest conjunctival retention. 

Overall, econazole micelles do not seem useful for promoting econazole transconjunctival transport; however, T micelles can be useful to increase drug concentration in the epithelium for the treatment of conjunctival mycosis. 

### 3.5. Dexamethasone

#### 3.5.1. Dexamethasone Solubility

The use of polymeric micelles had a relatively limited effect on dexamethasone solubility, compared to cyclosporine and econazole. The solubility obtained was about 0.5 mg/mL for T and TP and 1.1 mg/mL in the case of P (Table 4). The higher solubility in the presence of poloxamer 407 can be explained by the relatively low logP of dexamethasone (1.87), which probably has more affinity for the PPO block of poloxamer 407 compared to the highly lipophilic core of TPGS and by the higher percentage concentration of P when expressed by weight. The size and zeta potential of micelles were not affected by dexamethasone loading (Table 4).

#### 3.5.2. Dexamethasone Permeation across the Conjunctiva

**Figure 4:**
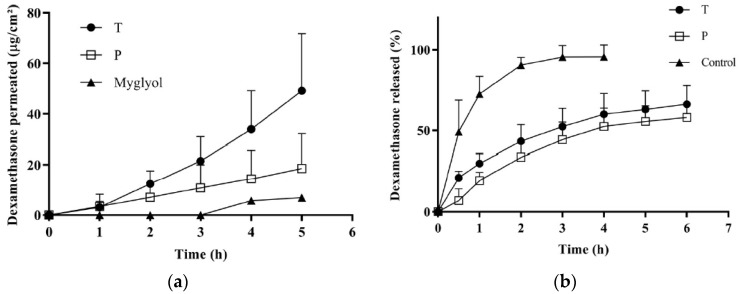
(**a**) Transconjunctival permeation profiles of dexamethasone from formulations T, P and from Miglyol^®^ dispersion; (**b**) dexamethasone release from T and P micelles and from a solution (EtOH:PBS 1:1).

Figure 4a illustrates the permeation profiles obtained from T, P and the control suspension in Miglyol^®^. The data indicate the good permeation properties of dexamethasone, probably thanks to its favorable partition coefficient and small size. Micellar solutions performed better than the control vehicle and, in agreement with the econazole data, the permeation from formulation T was significantly higher than the permeation from P. The apparent permeability coefficient (calculated between 4 and 5 h) was about three times higher for T compared to P, as well as the amount accumulated in the tissue (Table 5).

#### 3.5.3. Dexamethasone Release from Micelles

In order to investigate the reasons for the different behavior between P and T, the dexamethasone release profile from the micelles was studied. The results are reported in Figure 4b, compared with a reference solution in (EtOH:PBS 1:1), and show that T and P micelles were able to control dexamethasone release in a comparable manner. This indicates that the different performance of T and P cannot be due to a different release kinetic from the micelles.

#### 3.5.4. TPGS Transconjunctival Permeation and Mechanism of Enhancement

**Figure 5:**
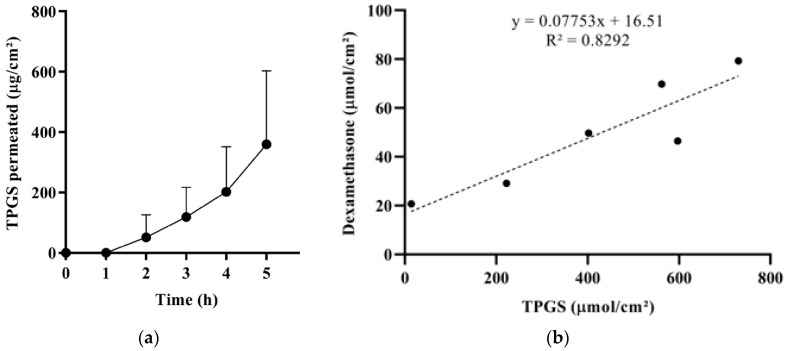
(**a**) Transconjunctival permeation profile of TPGS from T; the apparent permeability coefficient (calculated in the 4–5 h interval) resulted (1.32 ± 0.90) × 10^−6^ cm/s. (**b**) Correlation between the amount (µmol/cm^2^) of dexamethasone and the amount of TPGS permeated across the conjunctiva after 5 h (coupled data).

To further study micelles’ behavior, the permeation of TPGS from T formulation was measured (Figure 5a), and the amount (µmol/cm^2^) of dexamethasone that permeated after 5 h was plotted as a function of the amount (µmol/cm^2^) of TPGS permeated (Figure 5b). The result suggests that dexamethasone penetrates partially as a single molecule, independently of the presence of TPGS (see the significant y intercept) and partially in combination with TPGS (see the good linear correlation). The slope of the curve represents the dexamethasone:TPGS molar ratio found in the receptor compartment. This value (0.078) is similar to the dexamethasone:TPGS molar ratio in the donor compartment (0.075) and suggests the permeation of intact micelles. Indeed, some authors have demonstrated the capability of intact micelles to diffuse into ocular epithelial cells [64,65] and tissues [66].

The results obtained from the dexamethasone release test (Figure 4b) and TPGS permeation studies (Figure 5) suggest that the higher dexamethasone transconjunctival profile from T compared to P (Figure 4a) can be ascribed to a different micelle‒tissue interaction. Indeed, the lower performance of P may be due to the high viscosity of vehicle P, and thus to a lower mobility of poloxamer 407 molecules and micelles, trapped in the ordered face-centered cubic structure. The lower mobility could lead to less effective inhibition of efflux pumps, as well as a lower permeation enhancement capability of the unimer.

### 3.6. Effect of Micelle Application on the Conjunctival Tissue

**Figure 6:**
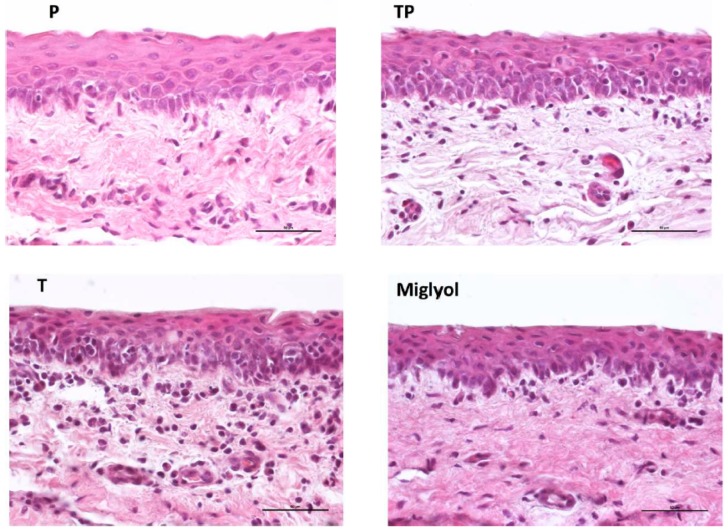
Histology of the conjunctival samples treated for 5 h with the different formulations. (Magnification 40×, bar = 50 µm).

The effect of micelles application (37 °C, 5 h), as well as of the oily vehicle Miglyol^®^, on the integrity of the conjunctiva was evaluated. The histological sections presented in Figure 6 show that the conjunctival epithelium retains its morphology and architecture, and is comparable to the nontreated tissue (Figure A2). In fact, the epithelial cell layers, consistent in number with the control sample (Figure A2), appear uninjured, with no visible gaps or edema, and their surface is regular and smooth.

The preservation of the conjunctival structure, studied by histology, is not sufficient to demonstrate the tolerability. It needs to be evaluated for both blank and loaded micelles using an irritation test in vivo. However, the evidence that poloxamer 407 is FDA-approved for ophthalmic application and TPGS is contained in medical devices present in the EU market (such as COQUN^®^ and Visudrop^TM^) or evaluated in clinical studies [67] represents an encouraging starting point. Furthermore, poloxamer and TPGS‒poloxamer mixed micelles [7,68] were nonirritant when evaluated using the chorioallantoic membrane test (HET-CAM assay), an in vitro organotypic assay commonly adopted in the preliminary assessment of the tolerability of ophthalmic formulations.

## 4. Conclusions

The data presented here demonstrate the potential of TPGS polymeric micelles in ocular drug delivery due to their ability to improve the solubility of the evaluated drugs and enhance their transport into and across the conjunctival epithelium. Indeed, the micellar formulations tested outperformed a solution/suspension in Miglyol^®^. 

The best-performing formulation was the one made of TPGS alone, probably because of the higher mobility of the micelles, a closer interaction with the conjunctival epithelium, and, possibly, the penetration of intact micelles. While, in the case of cyclosporine and dexamethasone (unionized drugs), TPGS micelles promoted both drug permeation across and retention inside the tissue, econazole nitrate (positively charged) was recovered only inside the conjunctiva. This may be due to its positive charge (promoting adhesion to the conjunctival surface) and to different drug localization in the polymeric micelle. 

Diseases of the surface of the eye (such as conjunctivitis, superficial infections, and dry eye disease) can take advantage of a high drug concentration in the epithelium. Furthermore, the penetration across the bulbar conjunctiva could in principle be exploited for posterior segment delivery (following the conjunctival-scleral pathway). However, systemic absorption can take place through the conjunctival blood and lymphatic vessels, especially ones localized in the palpebral conjunctiva, and the design of a drug delivery system for the targeting of the posterior segment requires further efforts to minimize systemic absorption and decrease the risk of off-target side effects. Furthermore, even if it has been demonstrated that drugs administered in the conjunctival sac can be found in the retina and vitreous, the clinical relevance of the amount delivered has been established in only a few cases. Despite these concerns, the possibility of a noninvasive targeting of the back of the eye represents an exciting perspective, and TPGS micelles could contribute to this achievement. Some aspects still deserve more attention, such as sterilization issues, tolerability, and micelles’ binding to the conjunctiva, which influence the ocular retention time and thus drug bioavailability. The latter requires the setup of a specific in vitro method before in vivo tests can be performed to evaluate the effective persistence into the conjunctival sac and the irritation potential.

## Figures and Tables

**Table 1:** Composition of the micellar formulations studied. The amounts of polymers reported, expressed both as mmol and mg, were added to 5 mL of water.

Formulation	TPGS		Poloxamer 407		Water
	mmol	mg	mmol	mg	mL
T	0.1	151.4	-	-	5
TP	0.05	75.71	0.05	632	5
PT	0.025	38.26	0.075	947	5
P	-	-	0.1	1264	5

**Table 2:** List of the formulations selected for transconjunctival permeation experiments, composition of the receptor medium, and of the mixtures used for drug extraction from tissue.

Drug	Donor		Receptor Phase	Extraction Mixture
	Vehicle	Drug Conc. (mg/mL)		
Cyclosporine	T	ss ^a^	PBS + TPGS 1 mM ^b^	CH_3_CN: 1% CH_3_COOH (87:13)
	Miglyol^®^ (solution)	5	PBS + TPGS 1 mM ^b^	
Dexamethasone	T ^c^	ss ^a^	PBS ^d^	CH_3_CN: water (35:65)
	P	ss ^a^	PBS ^d^	
	Miglyol^®^ (dispersion)	1.5	PBS ^d^	
Econazole Nitrate	TP	ss ^a^	PBS + TPGS 0.2 mM ^e^	CH_3_CN: pH 4 buffer (60:40)
	P	ss ^a^	PBS + TPGS 0.2 mM ^e^	
	T	ss ^a^	PBS + TPGS 0.2 mM ^e^	
	T	ss ^a^	PBS + 1% BSA ^f^	
	Miglyol^®^ (dispersion)	3.8	PBS + 1% BSA ^f^	

^a^ ss: saturated solution; see Table 4 for values. ^b^ Cyclosporine solubility: 290 ± 10 µg/mL. ^c^ In this condition, also the transport of TPGS across the conjunctiva was evaluated. ^d^ Dexamethasone solubility: 79 ± 4 µg/mL. ^e^ Econazole nitrate solubility: 91 ± 8 µg/mL; ^f^ Econazole nitrate solubility: 245 ± 29 µg/mL. BSA was precipitated before analysis by adding 260 µL of CH_3_CN and 40 µL of HClO_4_ to the receptor samples (300 µL); the recovery efficiency was evaluated and resulted >85%.

**Table 3:** HPLC-UV methods for the different analytes.

Parameter	Cyclosporine	Dexamethasone	Econazole Nitrate	TPGS
Column (Waters, Milford, MA, USA)	Nova-Pack C_18_ (150 × 3.9 mm, 4 µm)	Nova-Pack C_18_ (150 × 3.9 mm, 4 µm)	XTerra RP18 (4.6 × 100 mm, 3.5 µm)	C_18_ Simmetry 300 (250 × 4.6 mm, 5 µm)
Oven temperature (°C)	65	45	37	40
Mobile phase	CH_3_CN: water with TFA 0.1% (65:35 *v*/*v*)	CH_3_CN: water (35:65 *v*/*v*)	CH_3_CN: pH 4 acetate buffer (70:30 *v*/*v*)	MeOH: pH 5.8 acetate buffer (97:3 *v*/*v*)
Flow (mL/min)	1.6	1.0	1.0	2.0
Absorption wavelength (nm)	230	240	223	215
Retention time (min)	~5	~3	~2.5	~4

**Table 4:** Characteristics of blank and drug-loaded micelles. Micelle size reported is from DLS analysis; (n.d. = not determined).

**Blank**	**pH**	**Zeta Potential (mV)**	**Solubility (mg/mL)**	**Size (nm)**	**% Intensity**	**PdI**
T	6.00 ± 0.02 ^a^	−8.26 ± 4.03	-	13.86 ± 4.30	100.0%	0.132
TP	6.94 ± 0.01	−8.09 ± 1.87	-	19.93 ± 8.03	96.8%	0.205
P	7.22 ± 0.01	−3.32 ± 8.93	-	See Table S2		
**Cyclosporine**	**pH**	**Zeta Potential (mV)**	**Solubility (mg/mL) ^¶^**	**Size (nm)**	**% Intensity**	**PdI**
T	6.82 ± 0.07	n.d.	4.84 ± 0.45	12.96 ± 4.03	100.0%	0.091
TP	6.88 ± 0.02	−0.02 ± 0.20 ^e^	0.99 ± 0.23	20.31 ± 9.66	100.0%	0.192
P	7.04 ± 0.01	n.d.	0.57 ± 0.38	n.d.		
**Econazole Nitrate**	**pH**	**Zeta Potential (mV)**	**Solubility (mg/mL) ^#^**	**Size**	**% Intensity**	**PdI**
T	3.36 ^b^ ± 0.02	9.13 ± 1.48	3.69 ± 0.20	7.21 ± 2.15	100.0%	0.076
T ^c^	5.1 ^d^ ± 0.14	n.d.	n.d.	12.02 ± 3.58	100.0%	0.094
TP	4.23 ± 0.10	4.52 ± 1.68	4.15 ± 0.17	17.85 ± 8.50	99.5%	0.19
P	4.23 ± 0.10	−0.71 ± 1.48	3.79 ± 0.56	n.d.		
**Dexamethasone**	**pH**	**Zeta Potential (mV)**	**Solubility (mg/mL) ^#^**	**Size**	**% Intensity**	**PdI**
T	6.54 ± 0.16	−11.39 ± 2.94	0.59 ± 0.03	13.44 ± 4.29	97.7%	0.165
TP	6.94 ± 0.02	−10.55 ± 4.69	0.53 ± 0.09	21.39 ± 11.54	99.6%	0.222
P	7.10 ± 0.03	−10.99 ± 3.30	1.11 ± 0.10	n.d.		

^¶^ measured at 36 days; ^#^ measured at 30 days; ^a^ blank T micelles brought to pH 3.3 have a comparable size (11.7 ± 2.4 nm); ^b^ at this pH, the logD is 2.4 and the % of unionized form is about 0.08% [30]; ^c^ addition of NaOH 0.5 M; ^d^ at this pH, the logD is 4.4 and the % of unionized form is about 8% [30]; ^e^ from a previous work [7], sample dilution 1:20.

**Table 5:** Apparent permeability coefficient (cm/s) and drug retention in the conjunctiva after 5 h of contact with micelles and the Miglyol^®^ solution/dispersion.

Formulation	Cyclosporine		Dexamethasone		Econazole	
	Conjunctiva Retention (µg/cm^2^)	P × 10^−6^ (cm/s) ^a^	Conjunctiva Retention (µg/cm^2^)	P × 10^−6^ (cm/s) ^a^	Conjunctiva Retention (µg/cm^2^)	P × 10^−6^ (cm/s) ^a^
T	13.34 ± 15.92	0.46 ± 0.26	9.68 ± 4.92	3.26 ± 1.78 ^¶^	30.88 ± 7.93 ^#,b^	-
					30.32 ± 5.23 ^#,c^	-
TP	n.d.	n.d.	n.d.	n.d.	7.95 ± 2.32	-
P	n.d.	n.d.	3.53 ± 3.71 ^¶^	1.10 ± 0.60	5.41 ± 2.85	-
Miglyol^®^	<LOQ	-	4.96 ± 0.49	-	2.40 ± 0.44	-

^¶^ statistically different from P (*p* < 0.05); ^#^ statistically higher for comparison with TP, P and Miglyol^®^ (*p* < 0.05); ^a^ apparent permeability coefficient calculated from the 4–5 h flux; ^b^ pH 3.3; ^c^ pH 5.1; n.d., not determined.

## Supplementary Materials

The following are available online at https://www.mdpi.com/1999-4923/11/9/476/s1, Table S1: Validation parameters of the HPLC methods; Table S2: DLS analysis of P formulations at different dilutions; Table S3: Values of peak positions observed by small angle X ray scattering (SAXS) patterns analysis for formulation P at 25 °C. Figure S1. SAXS spectra of mixed TP micelles (50:50 mole ratio) prepared with different protocols.

## Appendix A

The method used for diffusion cell assembling is reported in Figure A1.

Figure A2 illustrates the histology of the porcine conjunctival tissue at different magnifications. With the lower magnification image, it is possible to see the forniceal and the bulbar conjunctiva, the underlying sclera, and its transition to the cornea, characterized by a different organization of the collagen fibers. In the 10× magnification it is possible to appreciate, below the epithelium, the substantia propria divided into a more superficial lymphoid layer and a deeper fibrous layer containing the vessels (arterioles and venules) [17]. The 40× magnification shows the conjunctival epithelium with 4‒6 cell layers.
