# Supplementary material for: Ex Vivo Conjunctival Retention and Transconjunctival Transport of Poorly Soluble Drugs Using Polymeric Micelles
Source: Pharmaceutics. 2019 Sep 14;11(9):476. doi: 10.3390/pharmaceutics11090476 (PMC6781556; doi:10.3390/pharmaceutics11090476)
Supplement: Supplementary file 1 [file pharmaceutics-11-00476-s001.pdf]

pharmaceutics

Supplementary Materials: Ex Vivo Conjunctival Retention and Transconjunctival Transport of Poorly Soluble Drugs Using Polymeric Micelles

Silvia Pescina, Leticia Grolli Lucca, Paolo Govoni, Cristina Padula, Elena Del Favero, Laura Cantù, Patrizia Santi and Sara Nicoli

Table S1. Validation parameters of the HPLC methods.

	Cyclosporine	Dexamethasone	Econazole Nitrate	TPGS
Linearity (μg/mL)[a]	0.25–5<br>2.5–50	0.05–1<br>1–100	0.1–5<br>5–100	2.5–50
LLOQ (μg/mL)<br>(RSD%; RE%)	0.25<br>(RSD% 1.72; RE% 12)	0.05<br>(RSD% 3.7; RE% 3.3)	0.1<br>(RSD% 9.03; RE% 19)	2.5<br>(RSD% 13.5; RE% 8.7)
LOD (μg/mL)	0.125	0.01	0.05	1
Recovery % from the ocular tissue	93.6 ± 5.2 [b,c]	100.0 ± 2.1 [b]	88.9 ± 2.6	nd

[a] Calibration curves at lower concentration were used for permeability studies, calibration curves at higher concentration were used for solubility evaluation. [b] The method was validated on corneal tissue, [c] From ref [7].

Table S2. Results of the DLS analysis of blank P formulations at different dilutions.

Dilution	Size (nm)	% Intensity	Z Average	PdI
1:10	6.6 ± 2.3	69.40%	76.19	0.29
	33.9 ± 13.3	29.00%		
	5023 ± 693	1.70%		
1:50	8.4± 2.9	81.70%	9.215	0.328
	568.4 ± 580.3	18.30%		
1:100	8.3 ± 2.5	69%	30.88	0.345
	470.7 ± 236.6	22.30%		
	4495 ± 956	7.2%		

Table S3. Values of peak positions observed by small angle X ray scattering (SAXS) patterns analysis for formulation P at 25 °C. The determination of the crystalline phase, face-centered cubic FCC, has been obtained from the qi/q* ratios. The first-order peak q* is not present in the pattern.

Peak Position (nm^{-1})	q_i/q^*
$q^* = 0.2125$	
0.368	√3
0.43	2
0.61	√8
0.7	√11
0.74	√12
0.85	√16
0.93	√19

The determination of the intermicellar distance d is given by q* (and the lattice parameter a_{FCC}) according to $d = \frac{\sqrt{6}}{4}\frac{2\pi}{q^*} = \frac{\sqrt{2}}{4}\, a_{FCC}$. The calculated distance was d = 18.1 nm, with a corresponding size of Pluronic micelles of the order of 13 nm.

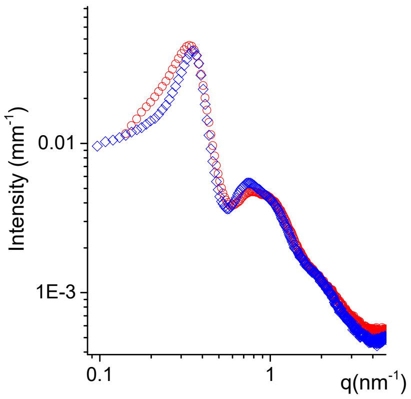

Figure S1. SAXS spectra of mixed TP micelles (50:50 mole ratio) prepared with different protocols. Dissolution of both powders in water (blue diamonds) and mixing of T and P solutions (red dots). Intensity profiles are similar in all the investigated q region.
